# γ-Secretase Modulators and APH1 Isoforms Modulate γ-Secretase Cleavage but Not Position of ε-Cleavage of the Amyloid Precursor Protein (APP)

**DOI:** 10.1371/journal.pone.0144758

**Published:** 2015-12-17

**Authors:** Christian B. Lessard, Barbara A. Cottrell, Hiroko Maruyama, Suraj Suresh, Todd E. Golde, Edward H. Koo

**Affiliations:** 1 Department of Neurosciences, University of California San Diego, La Jolla, California, United States of America; 2 Center for Translational Research in Neurodegenerative Disease, Department of Neuroscience, College of Medicine, University of Florida, Florida, United States of America; 3 Departments of Medicine and Physiology, Yong Loo Lin School of Medicine, National University of Singapore, Singapore, Singapore; National Center for Geriatrics and Gerontology, JAPAN

## Abstract

The relative increase in Aβ42 peptides from familial Alzheimer disease (FAD) linked *APP* and *PSEN* mutations can be related to changes in both ε-cleavage site utilization and subsequent step-wise cleavage. Cleavage at the ε-site releases the amyloid precursor protein (APP) intracellular domain (AICD), and perturbations in the position of ε-cleavage are closely associated with changes in the profile of amyloid β-protein (Aβ) species that are produced and secreted. The mechanisms by which γ-secretase modulators (GSMs) or FAD mutations affect the various γ-secretase cleavages to alter the generation of Aβ peptides have not been fully elucidated. Recent studies suggested that GSMs do not modulate ε-cleavage of APP, but the data were derived principally from recombinant truncated epitope tagged APP substrate. Here, using full length APP from transfected cells, we investigated whether GSMs modify the ε-cleavage of APP under more native conditions. Our results confirmed the previous findings that ε-cleavage is insensitive to GSMs. In addition, fenofibrate, an inverse GSM (iGSM), did not alter the position or kinetics of ε-cleavage position *in vitro*. APH1A and APH1B, a subunit of the γ-secretase complex, also modulated Aβ42/Aβ40 ratio without any alterations in ε-cleavage, a result in contrast to what has been observed with PS1 and APP FAD mutations. Consequently, GSMs and APH1 appear to modulate γ-secretase activity and Aβ42 generation by altering processivity but not ε-cleavage site utilization.

## Introduction

Accumulation of β-amyloid peptide (Aβ) aggregates in senile plaque is one of the pathological hallmarks of Alzheimer disease (AD), thus understanding the factors that regulate Aβ production, clearance, and aggregation has been a major focus of research [1]. The Aβ peptide is generated from sequential cleavages of the amyloid precursor protein (APP) first by β-secretase BACE [2–4], which generates the APP C-terminal fragment (CTF) of 99 amino (C99), followed by γ-secretase [5, 6]. The proteolytic cleavage of C99 by γ-secretase activity releases APP intracellular domain (AICD) into the cytosol and Aβ in the lumen/extracellular [7–9]. γ-Secretase is a macromolecular complex consisting of four proteins: nicastrin, APH-1, PEN-2 and presenilin (PS), the latter representing the catalytic subunit [10, 11]. Aβ peptides are heterogeneous, varying from 34–43 amino acids in length. Both heterogeneity at the N- and C-termini have been described with Aβ ending at different C-terminal residues having received most attention. Aβ1–40, or Aβ40 as commonly named, consists of 40 amino acids in length and is the most abundant species. Aβ42, though a minor species, is highly aggregation-prone, more neurotoxic in culture, and has been hypothesized to be the pathogenic trigger of AD [12]. In support of the latter concept, familial AD (FAD) mutations in *PSEN1* and *PSEN2*, and most FAD-linked mutations in *APP* genes consistently alter either the levels or profile Aβ peptides that are produced [13]. To date, more than 200 *PSEN1* FAD mutations have been identified and in virtually all mutations that have been analyzed, there is an increase in the ratio of Aβ42/Aβ40 peptides either by an increase in the absolute levels of Aβ42 or by a decrease in Aβ40 levels [14, 15]. The ratio of Aβ42/Aβ40 peptides can also be altered by APH-1, another member of the γ-secretase complex. Specifically, the APH1B isoform favors the production of longer amyloid species and consequently modifies the Aβ42/Aβ40 ratio.

**Fig 1:**
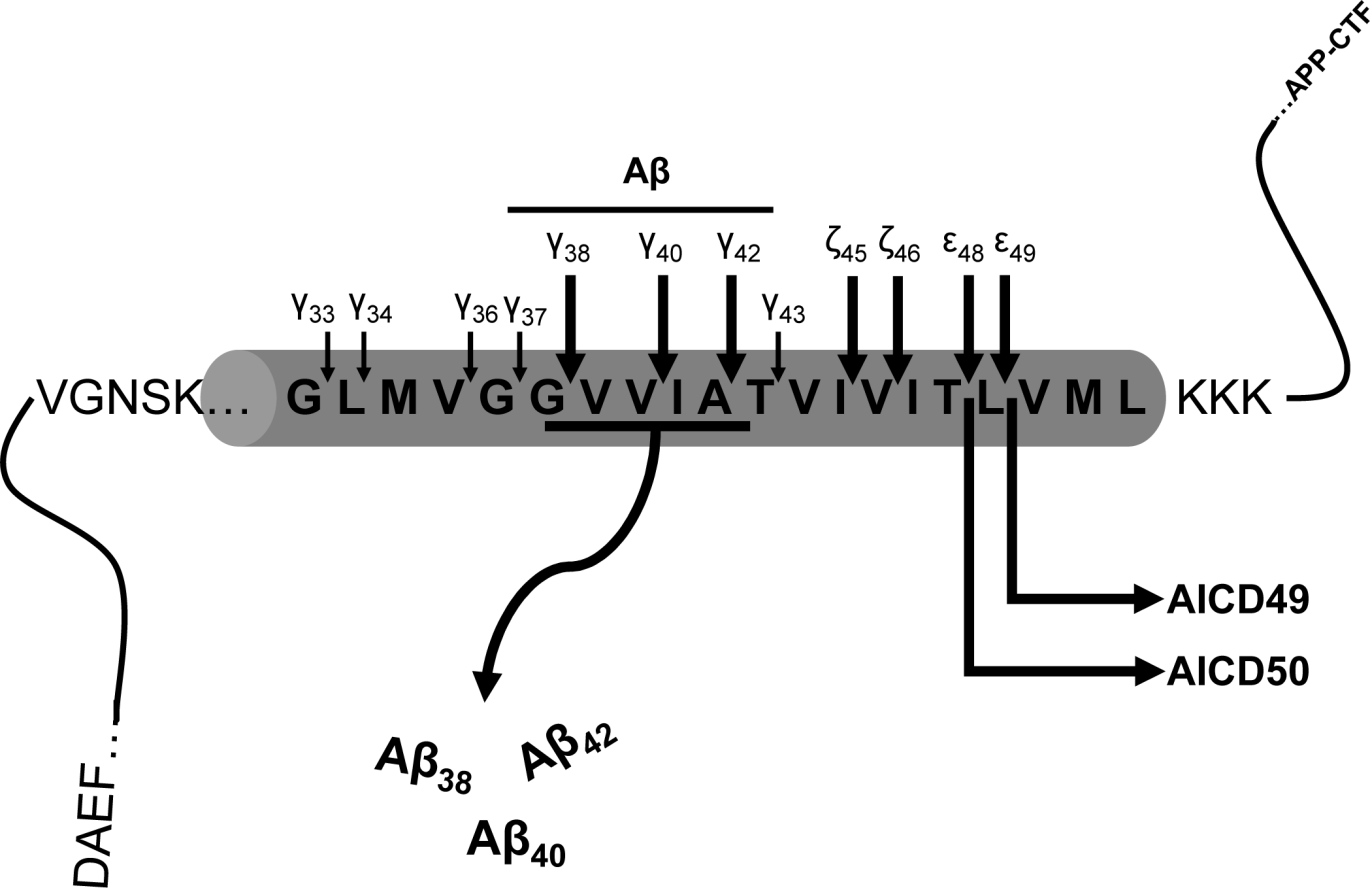
Schematic of proteolytic cleavages of APP by γ-secretase complex. In this model, ε-cleavage occurs first and releases AICD followed by γ-cleavage for Aβ secretion (adapted from Ihara and colleagues, [17]). The longer arrows sites indicated represent common Aβ species.

γ-Secretase cleavage has been described as “a proteosomal-like” activity due to its cleavage of many unrelated type 1 transmembrane protein substrates with little sequence selectivity and multiple cleavage products generated from each substrate [16]. Though the mechanism of by which γ-secretase cleaves substrates to generate multiple cleavage products remains unclear, a step-wise cleavage model first proposed by Ihara and colleagues provides an excellent framework for studying the generation of multiple APP cleavage products. In this model the first proteolytic step occurs at the ε-cleavage site situated near the cytoplasmic surface of the TMD to first release AICD (Fig 1). Subsequent processive cleavages at the ζ-position and proceeding every 3–5 amino acids result in γ-cleavage and release of Aβ peptides. However, the initial ε-cleavage is also imprecise, with major cleavages after Aβ48 or Aβ49 (APP positions 720 or 721, using APP770 numbering). It was further noticed that these two different initial cleavage positions appear to dictate Aβ peptides of different lengths. Thus, this model proposes, that after cleavage between Aβ49 and Aβ50, the dominant ε-site in wild type cells, the following peptides are generated in sequence: ε49-ζ46-γ43-γ40. In contrast, shifting cleavage one position to the N-terminus results in ε48-ζ45-γ42 [17, 18]. Accordingly, the most abundant AICD species is composed of 50 amino acids (AICD50), released after ε-cleavage following Aβ49 position (Fig 1). Consistent with this model of processive cleavage, FAD mutations that increase Aβ42 levels appear to perturb the position of ε-cleavage so that more AICD51 is generated [19, 20]. Thus, the ε and the γ-cleavages appear to work coordinately to generate the profiles of Aβ peptides that differ at the C-termini. However, this relationship between ε- and γ cleavage is not an absolute [21].

Inhibiting secretase activity to lower Aβ generation is one approach to reducing Aβ levels in brain. However, non-selective inhibition of γ-secretase activity carries the liability of inhibiting the proteolysis of many substrates of γ-secretase, such as Notch receptor. Indeed, mechanism based toxicity is almost certainly responsible for the adverse side effects seen in the γ-secretase inhibitor trials [22, 23]. However, it is clear that it may be possible to more selectively alter γ-secretase cleavage and avoid toxicities associated with pan-inhibition. Initially described as a property of a subset of non-steroidal anti-inflammatory drugs (NSAIDs), γ-secretase modulators (GSMs) preferentially lower Aβ42 production without impairing overall Aβ levels. Because GSMs do not block overall γ-secretase enzymatic activity, they also do not affect cleavage of a number of γ-secretase substrates that have been examined to date [24]. Since the initial description of that some NSAIDs act as GSMs, more potent second and third generation compounds have been reported and they generally fall into two classes. NSAIDs or NSAID-like compounds require carboxylic acid moiety for activity and these compounds generally follow the initial description where lowering of Aβ42 is accompanied by an increase in shorter peptides such as Aβ38 while sparing Aβ40 production. Non-acidic compounds based on, for example, the imidazole series, lower both Aβ40 and Aβ42 and typically increase both Aβ37 and Aβ38. In addition, compounds causing the inverse phenomena, i.e. raising Aβ42 while decreasing shorter Aβ peptides, termed inverse GSMs (iGSMs) have also been described. These iGSMs include fenofibrate and celecoxib. Finally, for unclear reasons, some presenilin mutations confer resistance to GSM activity [25–27]. In general, NSAIDs appear to bind substrate, i.e. APP, near the membrane interface [28]. However, later generation compounds, especially the non-acidic series, all appear to bind directly to the catalytic presenilin subunit of the γ-secretase complex [29]. Targeting of GSM to presenilin is consistent with earlier studies indicating an allosteric modulation of the complex by biochemical and morphological studies [30].

The precise mechanism action of GSMs remains unclear. GSMs can act noncompetitively to alter the conformation of substrate containing γ-secretase complexes [30–34]. And, as described above, GSMs have been shown to bind to either substrate or enzyme. Given the importance of the ε-cleavage event, several laboratories have examined the effects of GSM on this cleavage event and found that the overall levels of AICDs species 50 and 51 appeared unchanged upon GSMs treatment [29, 35, 36]. However, neither NSAID with GSM activity nor iGSM were examined in these studies. Accordingly, in this study, flurbiprofen, a NSAID with GSM activity, and fenofibrate, an iGSM, were thoroughly characterized and our results were consistent with the published reports, namely, no effects on ε-cleavage site. Further, there were no subtle alterations in the kinetics of ε-cleavage after fenofibrate treatment. Lastly, our results showed that APH1B, a component of the γ-secretase complex that increases Aβ42 levels, also did not modify ε-cleavage position. Therefore, our results appear to exclude alterations at ε-cleavage site as the mechanism by which GSMs, iGSM, and APH1B modulate the levels of Aβ42 generation.

## Materials and Methods

### Reagents

All other compounds and reagents were commercially available as follows: anti-rabbit IgG agarose beads and anti-mouse IgG agarose beads (American Qualex); flurbiprofen and β-Secretase Inhibitor IV (Calbiochem); fenofibrate (Sigma); complete protease inhibitor pellet (Roche); α-cyanohydroxycinnamic acid, trifluoroacetic acid, acetonitrile and phosphoramidon (Sigma). Monoclonal antibodies 82E1 (IBL) specific to N-terminus of Aβ, B436 (gift from Dr. Maria Kounnass) recognizing the N-terminal region of Aβ and polyclonal antibodies to recognizing the C-terminal 15 amino acids of APP (CT15) have been described previously [37, 38]. Anti-hemagluttin (HA) and and anti-Aβ17–24 (4G8) were purchased from Covance. Synthetic AICD50 and AICD51 peptides were custom synthesized by Peptide 2.0 Inc. GSM-1 was customized synthesized at the Mayo Clinic Jacksonville [39].

### Cell Lines and Culture Conditions

All cell lines were obtained from ATCC. Chinese hamster ovary (CHO) cell lines stably expressing WT-APP751 (7WD10), APP751-V717F mutation (7PA2), and wild type APP751 co-transfected with wild type PS1 L166P mutation have been previously described [40]. CHO 7WD10 cells were infected with retroviral vectors encoding wild-type PS1 or PS1-A79V and were selected with puromycin and stable pools of transfectants were analyzed without further clonal selection. CHO or HEK293T cells lines were grown in Dulbecco’s modified Eagle’s medium (DMEM) high glucose containing 10% fetal bovine serum, penicillin, and streptomycin. HEK-293T cells were transiently transfected by CaCl2 method. APH1A-HA or AHP1B-HA cDNA containing HA tag were stably transfected into APP expressing wild type cells (7WD10) via retroviral infection as above and selected by Zeocin resistance. APH1A-HA and AHP1B-HA cDNAs were a generous gift of Dr. Bart De Strooper.

### Cell-free assays for γ-secretase activity

AICD was generated *in vitro* from membrane preparations as previously described [41, 42]. In brief, cells were lysis in hypotonic buffer (10 mM tris HCl pH 7.4, 1 mM EDTA pH 8, 1 mM EGTA pH 8 and complete protease inhibitor) followed by 5 passages through a 27-gauge needle and 5 passages through a 30-gauge needle. The membranes were isolated from post nuclear supernatant and incubated in sodium citrate without CHAPSO to avoid any influence by detergent. Samples were incubated at 37°C in the presence of DMSO (control) or with GSMs (flurbiprofen 500 μM, GSM-1 1μM or fenofibrate 100 μM) for 4 hours and then centrifuged at 100,000 G for 30 mins. The supernatant was resuspended in sample buffer (50 mM Tris-HCl pH 6.8, 4% SDS, 2% β-mercaptoethanol, 0.01% coommassie blue) and resolved by on urea tricine-SDS-PAGE [43], transferred and immunoblotted with APP C-terminal antibody to detect AICD peptides of different lengths. Alternatively AICD samples were fractionated by Bis-Tris SDS-PAGE gel (NuPAGE, Invitrogen) to detect total levels. For Aβ levels produced from crude cell membranes *in vitro*, cells were pre-treated 16 hours with phorbol 12-myristate 13-acetate (10 μM) and NH_4_Cl (50 mM) [44]. Crude membrane preparations were processed as described above and centrifuged. The membrane pellets were resuspended in Tris-SDS-urea buffer (50 mM Tris-HCl pH 7.6, 8M urea and 1% SDS) and incubated at 37°C 1 h [45]. Following centrifugation, the pellets were resuspended in sample buffer, immunoblotted for Aβ with 82E1 antibody. Western blots were analyzed by densitometrically with Image J.

### Immunoprecipitation and Mass Spectrometry Matrix-Assisted Laser Desorption/Ionization Time-Of-Flight mass spectrometry (MALDI-TOF)

MALDI-TOF was performed from cell-free immunoprecipitates with the following modifications [46]. AICD produced from the membrane preparations were mixed with 1% NP40 buffer and complete protease inhibitor and incubated overnight with APP CT15 antibody and anti-rabbit IgG agarose beads. Aβ was immunoprecipitated from conditioned medium of CHO transfected cells with B436 antibody. AICD and Aβ immunoprecipitates were washed twice with 0.1% Tween-20, once with 0.03% Tween-20, once with 1 mM HEPES and then vacuum dried. Samples were eluted with 0.1% TFA 75% acetonitrile and mixed 1:1 with alpha-cyano-4-hydroxycinnamic acid matrix (Agilent) and then spotted on the MALDI target. Mass spectra were acquired in reflector positive mode at 10,000 shots per spectrum using single shot protection and a delayed extraction time of 420 ns. The area of the isotope pattern (isotopic cluster area) was used as a measure of apparent relative abundance and expressed as a percent of total. Results from three independent experiments were averaged for statistical analysis.

## Results

### Effect of GSM on ε-cleavage in wild type APP

**Fig 2:**
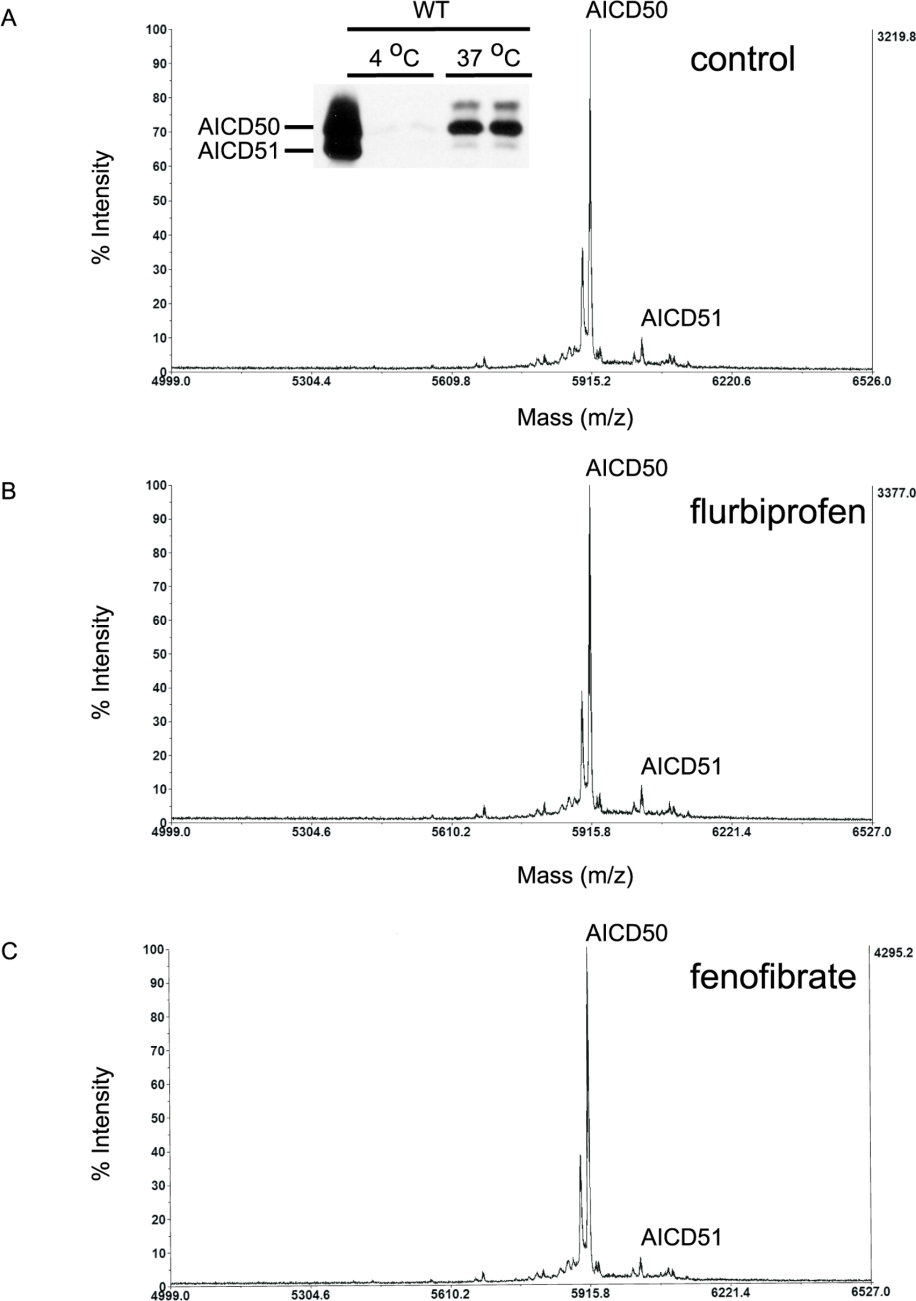
Neither flurbiprofen nor fenofibrate shifted ε-cleavage position in a cell-free assay by MALDI-TOF. Representative MALDI-TOF tracings of AICD peptides after incubation of crude membrane extracts of wild type APP expressing CHO cells at 37°C showed the presence of predominantly AICD50 fragment and minute amounts of AICD51 (A). Membrane preparations incubated with flurbiprofen (B) or fenofibrate (C) showed similar MALDI-TOF tracing as control. *Inset*: supernatant obtained from membranes shown in (A) incubated at 4°C and 37°C were analyzed by western blot. This confirmed that AICD50 was produced only following incubation at 37°C.

To determine if the modulatory activity of GSMs on γ-secretase activity subtly perturbs the position of ε-cleavage site, we determined the cleavage position of AICD fragment after drug treatment. Crude membranes from CHO cells stably transfected with wild type APP (7WD10) were first analyzed following drug treatment in cell free assay. Membrane preparations were incubated with flurbiprofen (500 μM) or fenofibrate (100 μM) and newly released AICD was analyzed by MALDI-TOF (Table 1). If the GSMs modulate ε-cleavage position to alter Aβ42/Aβ40 ratio, then the length of the AICD fragment will be correspondingly changed. Consistent with published reports, the major AICD species from wild type APP is a fragment of 50 amino acids in length (AICD 50) and released after cleavage between Aβ49 and Aβ50 or between the leucine and valine residues at positions APP720 and 721, respectively. AICD50 was detected by MALDI-TOF and immunoblotting (Fig 2A, inset). The immunoblotting results also confirmed that the AICD assayed was newly generated as no peptide was detected when the membranes were incubated at 4°C or blocked with γ-secretase inhibitor (data not shown). However, there was no detectable change in the profile of AICD fragments with either flurbiprofen or fenofibrate treatment as assessed by MALDI-TOF (Fig 2). Therefore, GSM failed to shift the position of ε-cleavage in wild type APP as assessed by MALDI-TOF.

**Table 1:** Molecular AICD species generated in cell free.

AICD species	calculated mass(m/z)	Observed mass (m/z)	Peptide sequence
51	6023.85	6024.73	LVMLKKKQYTSIHHGVVEVDAAVTPEERHLSKMQQNGYENPTYKFFEQMQN
50	5910.69	5910.66	VMLKKKQYTSIHHGVVEVDAAVTPEERHLSKMQQNGYENPTYKFFEQMQN
49	5811.56	5806.82	MLKKKQYTSIHHGVVEVDAAVTPEERHLSKMQQNGYENPTYKFFEQMQN

### AICD species in APPV717F mutation

**Fig 3:**
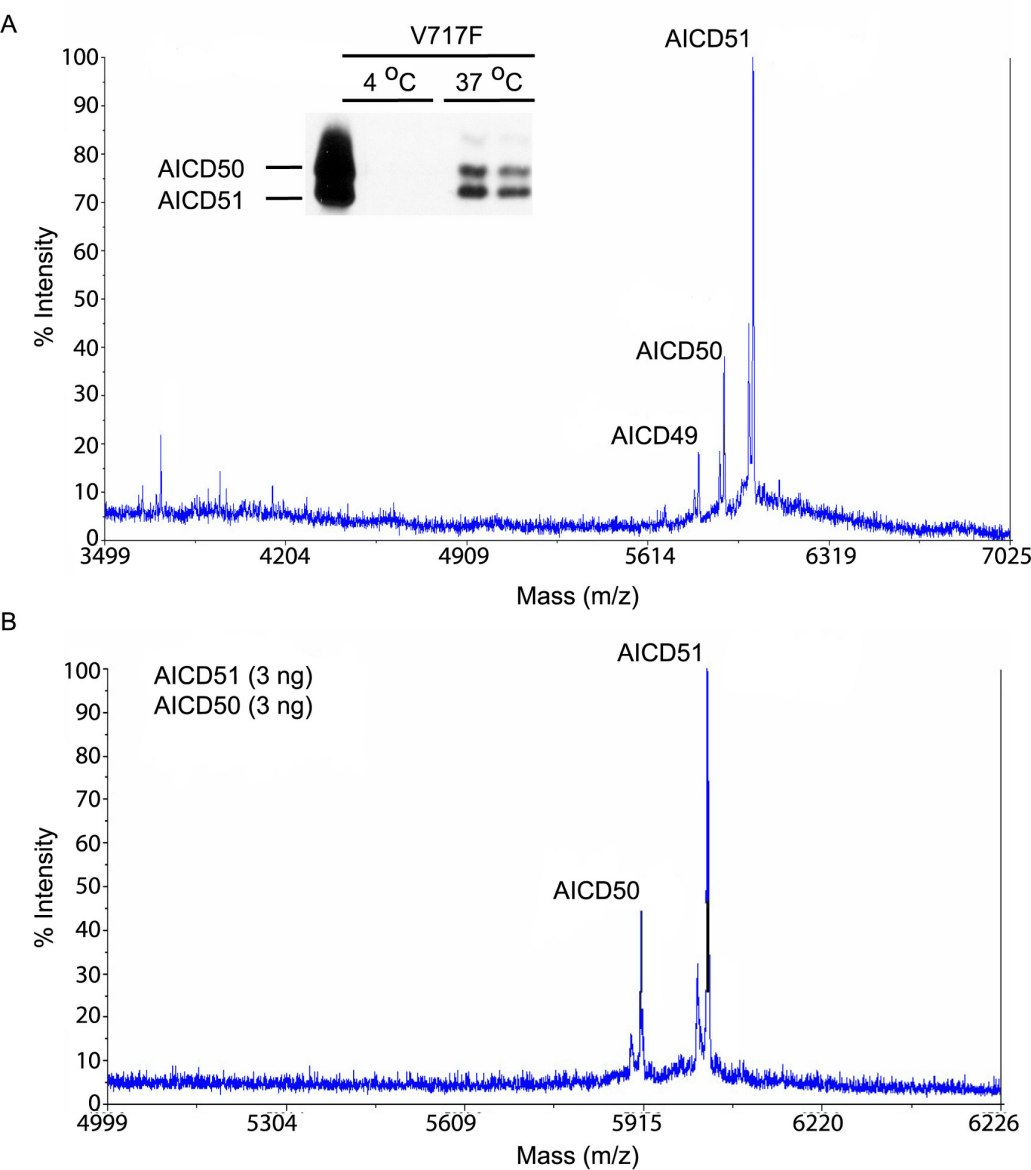
Different profile of AICD species from APP-V717F expressing CHO cells in cell free assay. MALDI-TOF tracings of AICD peptides after incubation of crude membrane extracts of APP-V717F expressing CHO cells at 37°C showed peaks representing AICD49, AICD50, and AICD51 (A). AICD51 showed the highest peak height of the three AICD species. In (B), equal amounts of synthetic AICD50 and AICD51 peptides were immunoprecipitated and analyzed by MALDI-TOF. AICD50 fragment did not ionize as efficiently as AICD51 fragment.

To confirm the preceding observation with APP V717F [20], a mutation known to shift the position of ε-cleavage, AICD fragments from membranes of CHO cells stably expressing APP V717F mutation (7PA2 cells) were analyzed. As reported previously [20], the predominant AICD fragment was AICD51 rather than AICD50 (Fig 3), thus, ε-cleavage has shifted one amino acid to the N-terminus (between threonine and leucine residues at APP positions 719 and 720, respectively). Western blotting of AICD fragments showed that the levels of AICD50 and AICD51 were comparable (Fig 3A inset) whereas in wild type APP, AICD50 was clearly the dominant species (Fig 2). Consequently, there was some disparity between the peak heights detected by MALDI-TOF and the AICD levels seen by Western blotting. To determine whether the ionization efficiency of the different AICD fragments is different, equal amounts of synthetic AICD50 and AICD51 peptides were analyzed together by MALDI-TOF. As can be seen, the AICD51 peak height was slightly more than two times higher than AICD50, confirming that the peptides were ionized quite differently (Fig 3B). As above, we did not detect any differences in AICD peaks after treatment with flurbiprofen or fenofibrate (data not shown).

### GSM and iGSM do not alter the ratio AICD50/AICD51 in wild type or mutant APP

**Fig 4:**
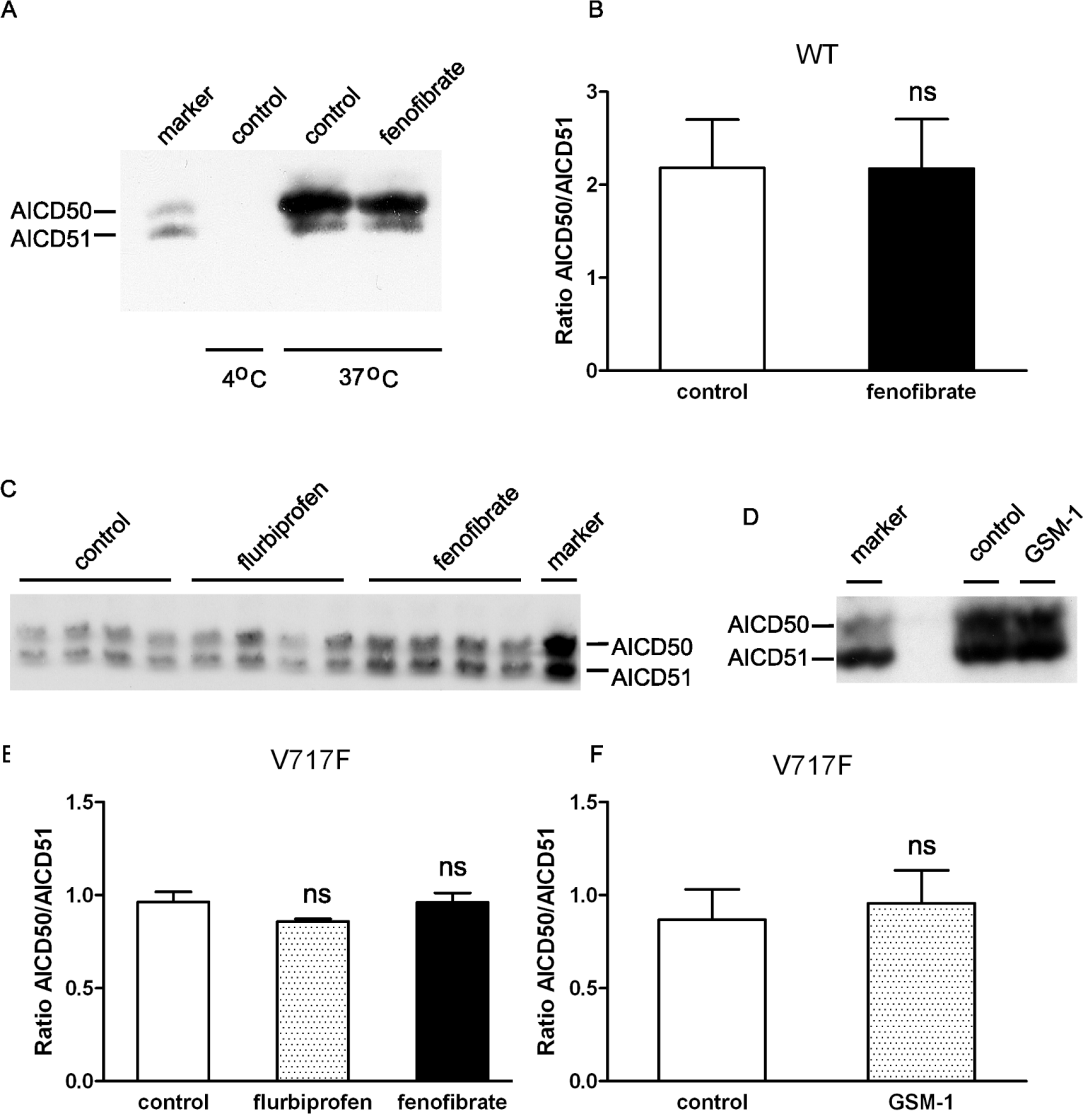
Flurbiprofen or fenofibrate did not shift ε-cleavage of wild type or mutant APP by western blot analyses. By immunoprecipitation and western blot analyses, neither flurbiprofen nor fenofibrate altered the length of AICD peptides generated from crude membranes from wild type APP (A, B) or APP-V717F (C, D) expressing CHO cells. Similar results were obtained with GSM-1 in APP-V717F expressing cells (E, F). Results were expressed as averaged ± standard error (B: n = 4, D: n = 3, and F: n = 3 experiments).

**Fig 5:**
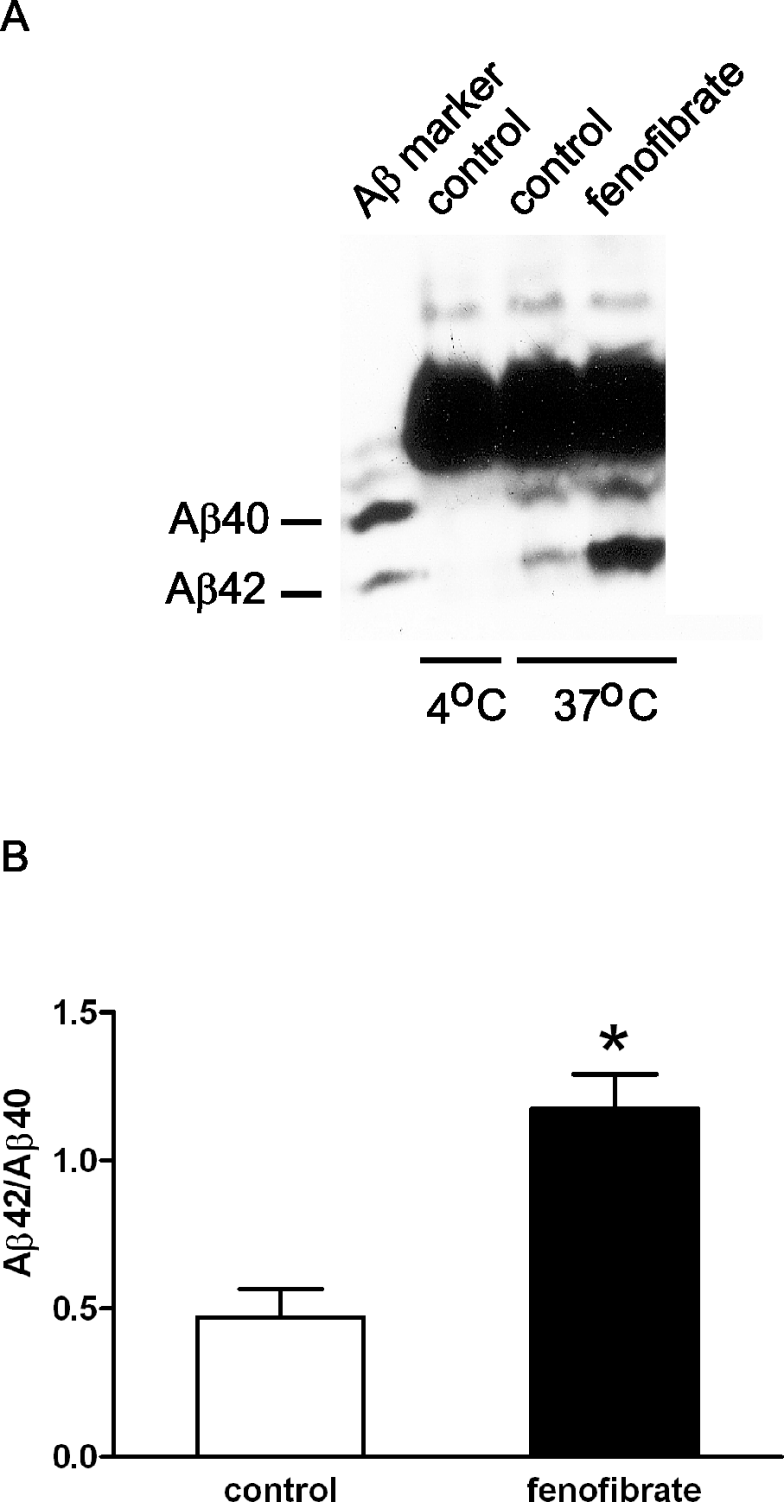
Fenofibrate demonstrated iGSM activity in cell-free assay. To confirm GSM activity, fenofibrate was added to crude membrane extracts during incubation and Aβ peptides recovered by immunoprecipitation and analyzed by western blotting using 82E1 antibody. In (A), addition of fenofibrate showed elevation of Aβ42 species. Shorter peptides such as Aβ38 and Aβ39 cannot be resolved due to overlap of APP C-terminal fragments in this gel system. Quantification of results from (A) is shown in (B) from the average of four experiments ± S.E. (* p < 0.05 two-tailed paired Student’s t test).

Given the unequal ionization of AICD peptides and to provide confirmation of the MALDI-TOF results, we next proceeded with semi-quantitative western blotting. CHO stable cell lines stably transfected with APP WT or APP V717F were analyzed for *de novo* AICD production from cell free membrane preparations treated with flurbiprofen (500 μM) or fenofibrate (100 μM). Accordingly, AICD50 and AICD51 fragments were fractionated on high resolution urea tricine SDS-PAGE to permit separation of these two species and analyzed by western blotting. Consistent with the MALDI-TOF results, no significant changes in the levels of AICD50 and AICD51, expressed as a ratio of the two species, were detected after fenofibrate treatment of membranes from APP WT expressing cells (Fig 4A and 4B) or APP V717F expressing cells (Fig 4C and 4D). Similarly, as reported, no significant changes in AICD fragments were detected following treatment with GSM-1, a second generation of GSM with high potency (Fig 4E and 4F). Thus, by two different assays, GSMs do not appear to alter the position of ε-cleavage site to modulate Aβ42 levels. Finally, given the lack of changes in ε-cleavage site, it was important to ascertain that there was in fact modulation of Aβ42 levels. Indeed, fenofibrate treatment significantly increased Aβ42 levels from crude membrane preparations without any apparent effects on Aβ40 levels (Fig 5A and 5B). Aβ38 cannot be resolved cleanly because of overlap with APP C-terminal fragments on this gel system.

### Fenofibrate does not affect γ-secretase kinetic for C99 substrate

**Fig 6:**
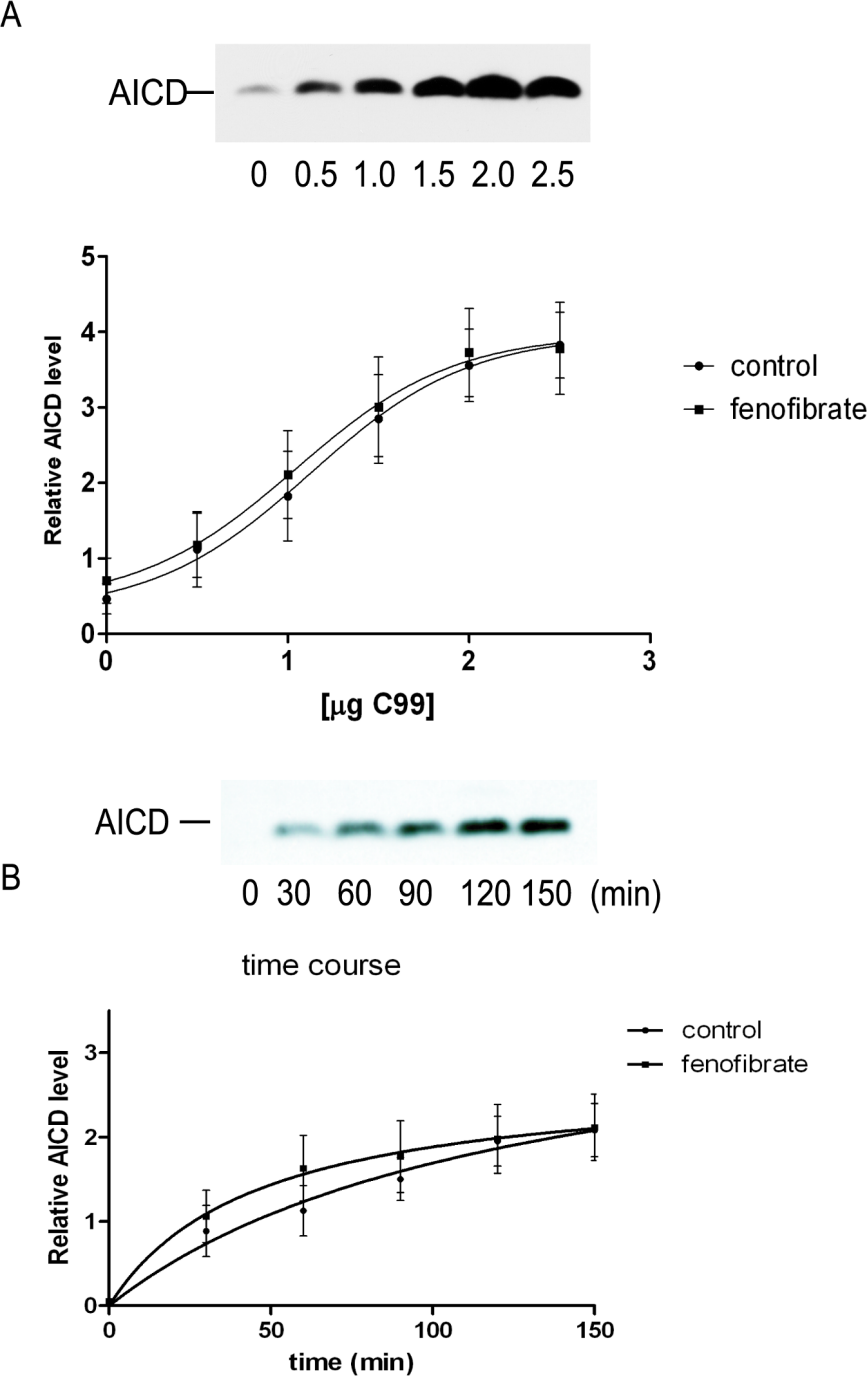
Kinetics of γ-secretase cleavage was not altered by fenofibrate in cell free assay. Levels of AICD were assessed by immunoblotting of HEK293T cells transfected with increasing quantity of APP C99 cDNA (A). The amounts of AICD generated from membranes treated with or without fenofibrate were the same regardless of starting amounts of transfected C99 cDNAs. (n = 6; averages ± S.E.). In (B), the levels of newly generated AICD were assayed from cells transfected with the same amount of C99 cDNA (1.5μg) and measured as a function of time. There was no change in the kinetics of AICD generation after treatment with fenofibrate (n = 6; averages ± S.E.).

A recent report showed that a number of PS1 FAD mutations decreased overall γ-secretase activity, thus reducing both Aβ40 and AICD levels [47], and this was also reflected by diminished kinetics of ε-cleavage activity. Consequently, we next asked whether fenofibrate might subtly alter the kinetics of ε-cleavage as seen PS1 FAD mutations. To investigate this possibility, a dose-response study on AICD generation was next performed in in HEK293T cells transfected with increasing amounts of APP C99 cDNA (to maximize AICD production) in the presence or absence of fenofibrate. Levels of newly generated AICD were proportional to the amount of transfected C99 cDNA (Fig 6A). However, there was no change in AICD levels after fenofibrate treatment as compared to control membranes (Fig 6A). To analyze the kinetics of AICD release from C99 substrate, the same quantity of C99 cDNA (1.5 μg) was transfected in HEK293T cells and membrane preparations were subsequently incubated for different time periods with and without drug treatment. As shown in Fig 6B, the release of AICD as a function of time was not altered in the presence of fenofibrate. In sum, fenofibrate, as the prototypic iGSM, did not perturb the kinetics of AICD release from C99.

### PS1 and APP FAD linked mutation modulate ε-cleavage

**Fig 7:**
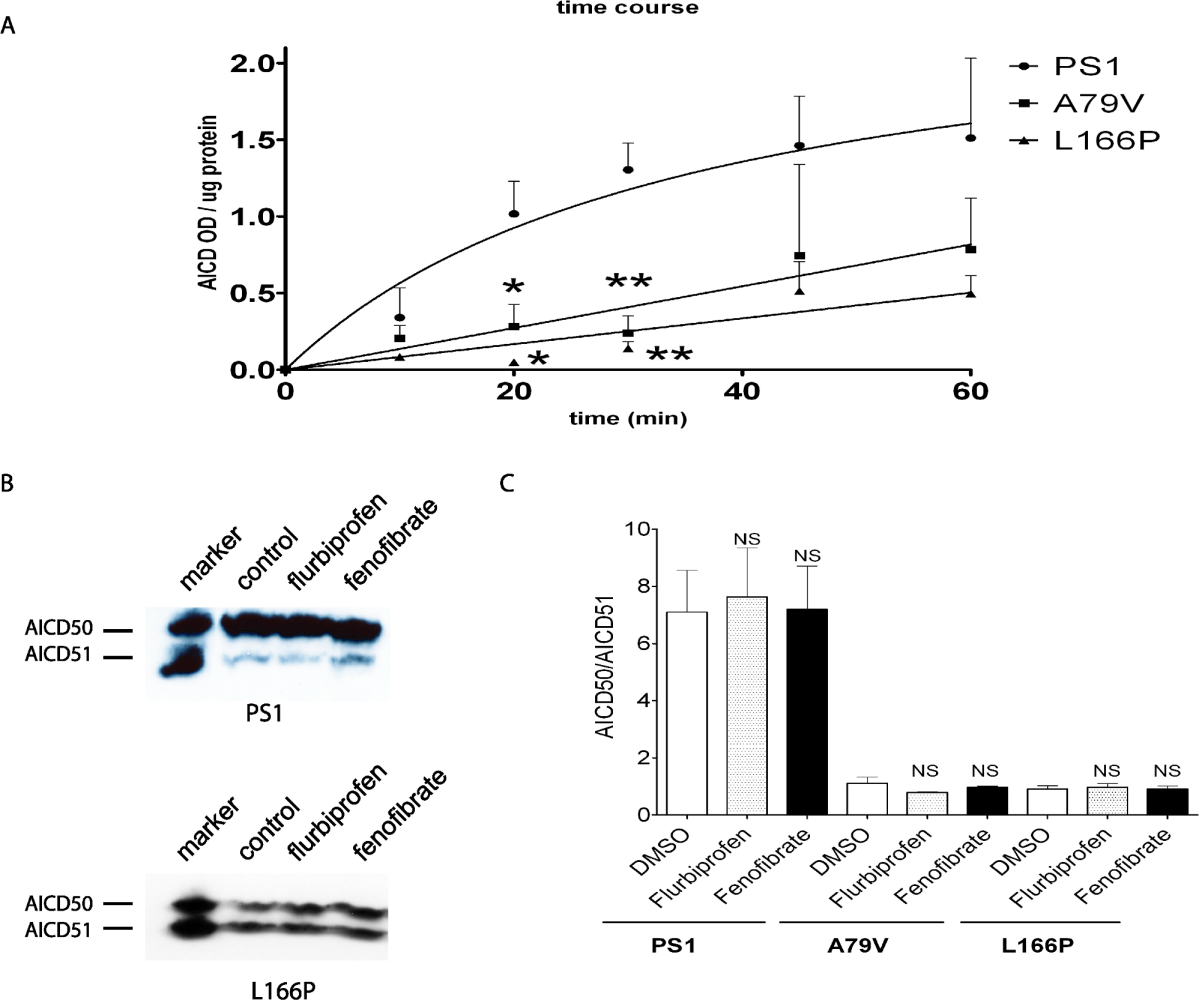
FAD-linked PS1 mutation showed reduced γ-secretase activity and also shifted position of ε-cleavage. CHO cells stably expressing wild type PS1 or A79V or L166P mutations were analyzed form AICD production. (A) Both PA1 mutations reduced γ-secretase activity as determined by the rate of AICD generated from crude cell membranes up to 60 minutes (n = 3; averages ± S.E). (B) By immunoprecipitation and western blotting, levels of AICD50 and AICD 51 were unchanged after addition of flurbiprofen or fenofibrate. (C) Quantification of results from (B) showed no significant changes in AICD50/AICD51 ratios after drug treatment (n = 3, “NS”: p > 0.05 one-way ANOVA; averages ± S.E.).

It has been shown previously that a subset of PS1 mutations are resistant to GSM treatment [48, 49]. We therefore wondered whether this unexplained resistance to GSM modulation might have altered ε-cleavage in some fashion by assessing two PS1 mutations: L166P, which is resistant to GSM, and A79V, which is sensitive to GSM treatment. Because it is has been reported that PS1 FAD mutants can reduce the level and kinetics of AICD production [47], we first performed a study on the time course of AICD generation from cells expressing these two mutations. By western blotting analyses, the levels and kinetics of AICD production level were indeed lower in membranes derived from PS1 A79V and L166P expressing cells as compared to wild type PS1 (Fig 7A). To determine whether GSM resistance observed in some PS1 mutations altered ε-cleavage position, CHO cells transfected with PS1 L166P or A79V mutation were analyzed. As seen, the ratio of AICD50/AICD51 fragments was not affected by flurbiprofen or fenofibrate treatment, just as seen in wild type PS1 (Fig 7B and 7C). However, as expected, there was an increase in the levels of Aβ42 peptide in the presence of PS1 L166P mutation (Fig 7B). Altogether, PS1 FAD mutations can reduce the efficacy of ε-cleavage and produce AICD species of different lengths but precise cleavage position was not altered by GSMs.

### APH1B increases Aβ42 secretion without shifting ε-cleavage

**Fig 8:**
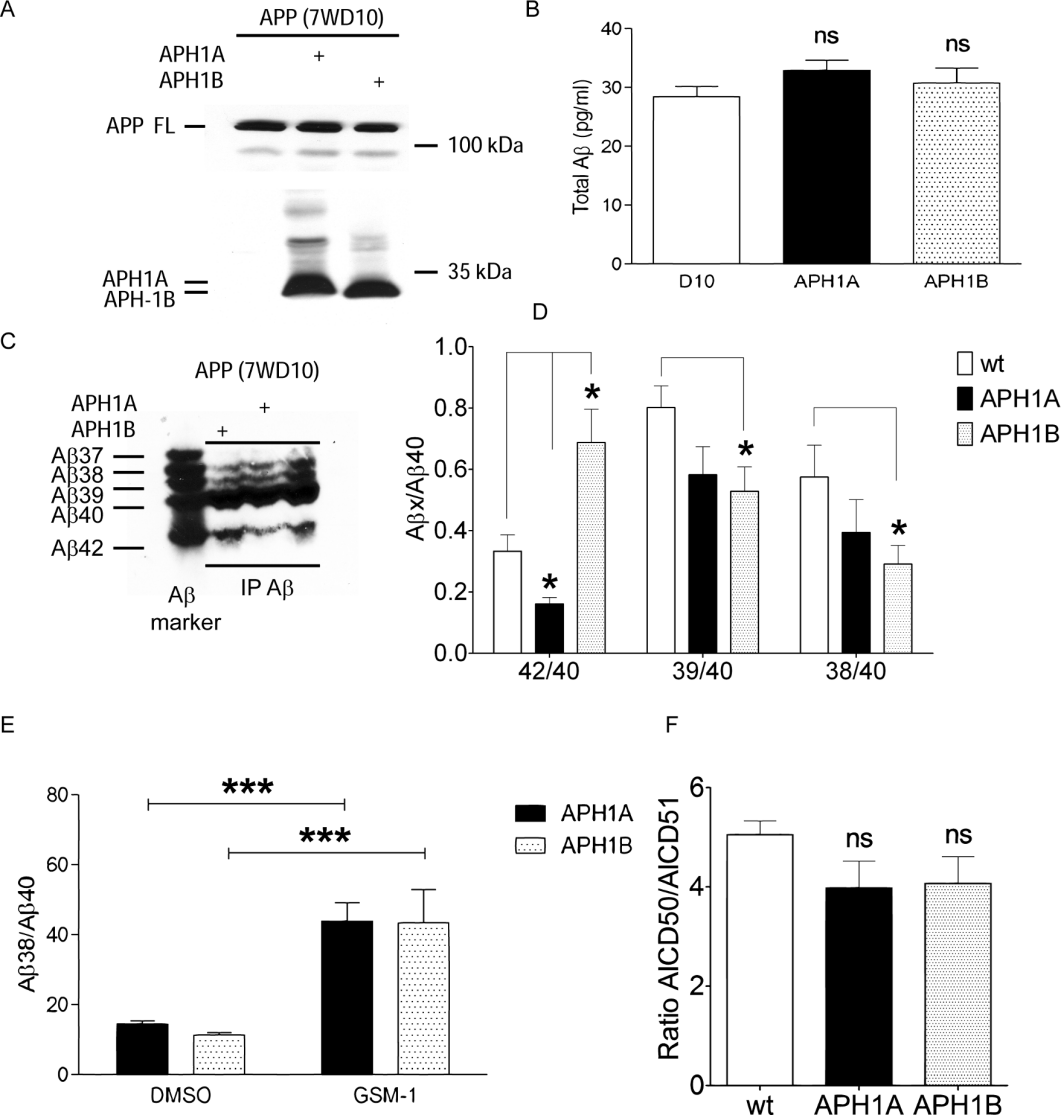
Effects of APH1A and APH1B on APP processing. (A) Comparable expression of APH1A or APH1B in CHO cells overexpressing wild type APP (7WD10) cells after stable transfection as detected by anti-HA antibody. (B) Expression of APH1A or APH1B had no effect on total Aβ levels as assessed by ELISA. (C) Representative western blot analysis of Aβ peptides from APH1A or APH1B expressing cells showing altered profile of Aβ peptides. (D) Quantification of Aβ levels of CHO cells expressing APH1A or APH1B shown in (C). Note the significant alterations in the ratios of Aβ42/40 as well as shorter Aβ species. However, Aβ42/40 ratios were altered in opposite directions following APH1A as compared to APH1B expression (n = 3; * p < 0.05, Repeated Measures ANOVA Tukey's Multiple Comparison Test; averages ± S.E.). (E) GSM-1 increased Aβ38 levels in CHO cells overexpressing either APH1A or APH1B (n = 3; *** p<0.001, Repeated Measures ANOVA Tukey's Multiple Comparison Test; averages ± S.E.). (F) No changes were seen in the ratio of AICD50/51 peptides quantified from crude membranes of CHO cells overexpressing either APH1A or APH1B and (n = 3, averages ± S.E.).

A previous study reported that expression of the APH1B isoform increased longer Aβ species [50]. Consequently, we investigated whether overexpression of APH1A or APH1B isoforms shifted ε-cleavage of APP in cell free assay similar to PS1 mutations as might be predicted given that it is integral to the γ–secretase complex. APH1A or APH1B was stably expressed in CHO cell line stably expressing wild type APP cell line (7WD10) (Fig 8A) without affecting the levels of total Aβ (Fig 8B). As anticipated, APH1B expression increased but APH1A decreased the Aβ42/Aβ40 ratio of secreted Aβ species (Fig 8C and 8D). In addition, APH1B expression decreased Aβ39/Aβ40 and Aβ38/Aβ40 ratios as compared to the wild type control while a non-significant trend was seen following expression of APH1A. Further, expression of APH1A or APH1B did not blunt the effects of GSM-1 as there was still a significant rise in Aβ38 levels after drug treatment (Fig 8E). However, like before, the expression of APH1A or APH1B was not accompanied by any changes in AICD50/AICD51 ratios, suggesting that modulation of Aβ42 levels by APH1B, unlike FAD mutations, is not linked to the position of ε-cleavage (Fig 8F). By modulating Aβ42/Aβ40 ratio without modulating ε-cleavage, APH1A and B appear to mimic the effects of GSMs rather than PS1 mutations. Taken together, through multiple analyses, the effects of GSM or iGSM on Aβ42 generation do not appear to be mediated at the level of ε-cleavage and this was not altered in the presence or absence of several FAD mutations or by expression of APH1B.

## Discussion

In this study, we asked whether GSMs and iGSM shift ε-cleavage as a potential mechanism through which the level of Aβ42 peptides are reduced. Our results showed that GSMs did not alter the position of ε-cleavage in cells expressing wild type APP or PS1, nor in cells overexpressing FAD associated APP or PS1 mutations. These results were obtained from both MALDI-TOF and immunoprecipitation/western blotting analyses of AICD species generated under cell free conditions. Significantly, GSM treatment was not associated with any subtle alterations in the kinetics of ε-cleavage, a reduction of which has been reported in the setting of PS1 mutations. Consistent with previous report, our results confirmed that overexpression of APH1A or APH1B altered Aβ42/Aβ40 ratios in opposite directions. Interesting, in neither condition was there a change in the position of ε-cleavage, indicating a mechanism unlike that seen in APP or PS1 mutations. Further, overexpression of APH1B did not impact the ability of GSM-1 to modulate γ-secretase cleavage. In sum, our study excludes a subtle action at the ε-cleavage site as a mechanism by which Aβ42 levels are preferentially reduced by GSM, iGSM, or APH1.

**Fig 9:**
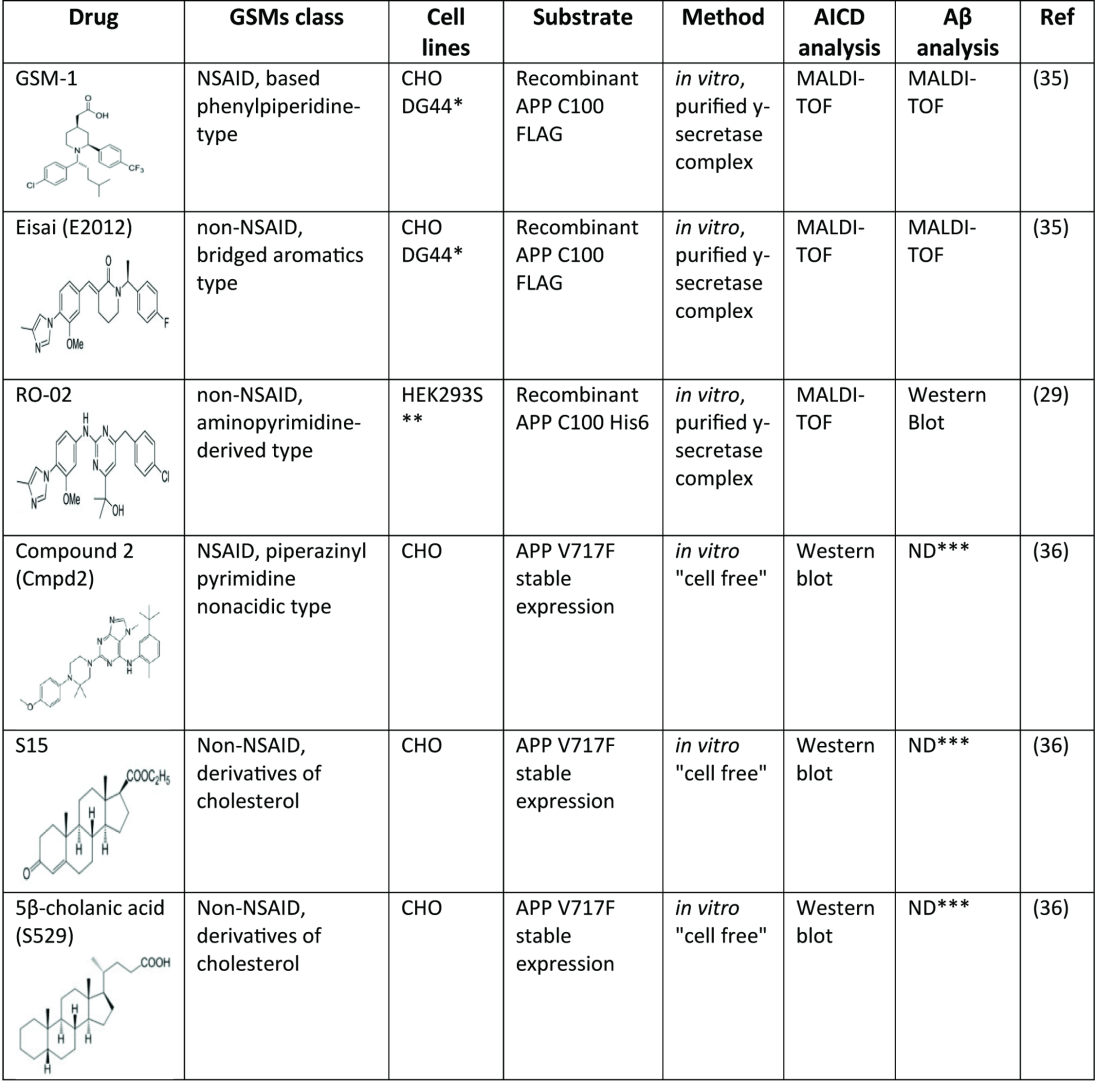
GSMs investigation, method and analysis type performed on AICD and Aβ.

First generation GSMs represented by a subset of NSAIDs lower Aβ42 and increase shorter Aβ peptides, such as Aβ37 and Aβ38 without altering Aβ40 levels. More potent second generation GSMs have demonstrated higher heterogeneity of action whereby both Aβ42 and Aβ40 levels may be reduced, but total Aβ levels remain unchanged. While NSAID based carboxylic GSMs appeared to bind APP in the Aβ region, second generation GSMs and non-acid GSMs bind PS1. This interaction is presumed to cause allosteric modification in γ-secretase activity to subtly perturb the profile of Aβ peptides that is generated. First proposed by the Ihara lab, the processivity model of sequential cleavage and release of tri- and tetra-peptides from APP by γ-secretase complex suggests that the initial ε-cleavage largely predicts the ratio of Aβ42/Aβ40 that will be produced. In particular, APP and PS1 mutations that enhance the Aβ42/Aβ40 ratio consistently changed the position of ε-cleavage in favor of longer AICD51 peptide over the AICD50 peptide that is predominant from wild type cells. In work from Okochi and colleagues, GSMs appear to accelerate the processivity of γ-secretase cleavage, thus allowing the generation of shorter Aβ peptides while reducing Aβ42 isoforms [51]. Consistent with this concept, several laboratories have reported that GSMs do not alter the position of ε-cleavage (Fig 9). Our results complement these studies using complementary methods as well as examining iGSM and the contribution of APH1 to modulating Aβ42 levels.

Consistent with previous reports, cell-free assays with crude membrane extracts from CHO cells transfected with wild type APP (7WD10 cells) showed a single dominant peak representing AICD50 in MALDI-TOF mass spectrometry [20]. As predicted, CHO cells expressing APP V717F mutation (7PA2 cells) showed multiple AICD species representing AICD49, AICD50 and AICD51 fragments. However, AICD species derived from membrane extracts of cells expressing full length wild type APP are notably different from cell free assays using recombinant APP C99 substrate reported by others. In the latter instance, recombinant C99 substrate produced AICD50 and AICD51 of comparable peak heights by mass spectrometry [29, 35, 52] whereas primarily AICD51 was produced from recombinant C99-V717F [35, 52]. Aside from being N-terminally truncated, recombinant C99 substrate is typically solubilized into membranes rather than a product of normal cellular processing. Taken together, these results suggest that the initial γ-secretase substrates used in cell-free assays may artefactually influence ε-cleavage of APP to generate different profiles of AICD species. This finding is not surprising in view of the pH dependency of ε-cleavage under cell free conditions as demonstrated previously [19].

MALDI-TOF is an invaluable method to detect small peptides in complex mixtures and has been especially informative in analyzing Aβ peptides in multiple settings. However, because unequal ionization efficiency may occur, peaks heights do not always correlate with peptide concentration. For example, Aβ42 is poorly ionized as compared to Aβ40, and in this study, we observed that AICD51 was much more readily ionized from the matrix than AICD50. This unequal ionization was confirmed by western blotting wherein approximately equal levels of AICD50 and AICD51 were present (Fig 3) but not reflected in the MALDI-TOF spectrometry. In this regard, most of the studies described in Fig 9 relied on mass spectrometry to assess the levels of various AICD fragments and thus the present study using western blotting complements these recent reports. The lack of any changes in AICD50/AICD51 ratio after treatments with GSMs or iGSM is entirely consistent with previous reports with others GSMs, such as RO-02 [29], E2012 [35], GSM-1 [35], Compound 2 [36], S15 and 5β-Cholanic acid [36]. In sum, it is safe to conclude that the modulation of Aβ species induced by a GSM or iGSM is not initiated at the ε-secretase cleavage step.

PS1 FAD mutations have been shown to result in partial to near complete loss of γ-secretase activity [13]. This can be seen in the cleavage of both APP and other substrates, such as Notch receptor. In particular, PS1 mutations such as Y115H, M139V, L166P, I213T, G384A and ΔE9, decreased ε-cleavage, resulting in a net reduction of AICD generation as compared to wild type PS1 [47]. In addition, the decrease in AICD production is accompanied by altered kinetics of ε-cleavage leading to diminished rapidity of AICD generation. Consistent with these observation, our results showed that two PS1 mutations, A79V and L166P, when stably expressed in CHO cells, demonstrated a significant reduction in the kinetics of ε-cleavage, as seen in a time course study of AICD release from cell free membrane preparations. However, this same impairment was not seen following fenofibrate treatment. Therefore, while the increase in Aβ42/Aβ40 ratio after iGSM treatment is reminiscent of PS1 FAD mutations, there was however no effect of fenofibrate on the kinetics of AICD production, unlike PS1 mutations.

In addition to iGSM, expression of the APH1B also resulted in higher levels of Aβ42 while reducing Aβ40 and shorter peptides [50]. Our results showed that stable expression of APH1A or APH1B in CHO cells modulated Aβ42/Aβ40 levels as compared to untransfected control cells. By increasing Aβ42/Aβ40 ratio, we hypothesized that APH1B subunit preferentially increases AICD51 levels similar to PS1 or APP FAD mutations. However, contrary to previous experience with PS1/APP FAD mutations, there was no significant modulation in ε-cleavage position as represented by AICD50/AICD51 ratio. This finding suggested that the mechanism by APH1 isoforms alter Aβ42 levels is different from that shown for APP and PS1 mutations. And while the magnitude of change in Aβ42/Aβ40 ratio is less than that seen with GSMs, overexpression of APH1 isoforms do have some resemblance to the effects of GSMs. Whether these activities of APH1 share similar molecular mechanisms as GSMs is an issue not addressed in the present study.

In conclusion, the findings in this study confirm recent reports that first generation GSMs such as flurbiprofen and second generation GSMs such as GSM-1 do not modulate ε-cleavage of APP, thus arguing that these compounds reduce Aβ42 production at a step following the initial ε-cleavage has been proposed. iGSM also failed to perturb ε-cleavage position. Interestingly, γ-secretase modulation is not only drug-specific but also influenced by γ-secretase complex itself, as exemplified by altering APH1 isoform levels. In the latter case, our studies also failed to detect any changes in ε-cleavage position. A recent computational study demonstrated that generation of Aβ40 is more energetically favored than Aβ42 [53], consistent with the ~ 9:1 ratio of Aβ40 to Aβ42 produced under basal conditions. However, this does not explain why the ε-cleavage favors the generation of AICD50 over other AICD species, a step that appears to determine in large part the subsequent production of Aβ40 species. Thus, understanding how GSM and APH1 influence the levels of Aβ42 and Aβ40 peptides may hold promise in deciphering the molecular mechanisms of γ-secretase cleavage of APP.

## Abbreviations

Aβ: amyloid β
AD: Alzheimer’s Disease
APP: Amyloid Precursor Protein
CHO: Chinese hamster ovary
HEK: human embryonic kidney
MALDI-TOF: matrix-assisted laser desorption ionization time of flight
NSAIDs: non-steroidal anti-inflammatory drugs
PS: Presenilin
TMD: transmembrane domain
